# Primary Health Care nurses’ role in treating Lower Urinary Tract Dysfunction^*^

**DOI:** 10.1590/1980-220X-REEUSP-2023-0146en

**Published:** 2024-03-01

**Authors:** Gisela Maria Assis, Nayara dos Santos Rodrigues, Franciele de Freitas de Oliveira, Camilla Pinheiro Cristaldi da Silva, Drielle Fernanda Arruda, Ana Carolina Silvy Nunes, Gisele Martins

**Affiliations:** 1 Universidade de Brasília, Departamento de Enfermagem, Brasília, DF, Brazil.; 2 Universidade Federal do Paraná. Curitiba, PR, Brazil.; 3 Pontifícia Universidade Católica do Paraná. Curitiba, PR, Brazil.; 4 Universidade Federal do Paraná, Programa de Residência Multiprofissional. Curitiba, PR, Brazil.; 5 Centro Universitário Estácio de Sá. Florianópolis, SC, Brazil.

**Keywords:** Education, Nursing, Lower Urinary Tract Symptoms, Primary Health Care, Enterostomal Therapy, Educación en Enfermería, Síntomas del Sistema Urinario Inferior, Atención Primaria de Salud, Estomaterapia, Educação em Enfermagem, Sintomas do Trato Urinário Inferior, Atenção Primária à Saúde, Estomaterapia

## Abstract

**Objective:**

To understand Primary Health Care nurses’ role in treating Lower Urinary Tract Dysfunction.

**Method:**

Cross-sectional multi-methodological research, composed of quantitative and qualitative steps, independently and sequentially. Data collected remotely, through a questionnaire and focus group, analyzed using descriptive statistics and thematic analysis by Braun and Clarke, respectively. The project was approved under Opinion 22691119.0.0000.0030.

**Results:**

A total of 145 nurses participated in the study in the quantitative step and 20 in the qualitative step, working in Primary Health Care in Brazil. Of the 93.1% nurses who reported having already cared for people with Urinary Tract Dysfunction, only 54.4% provided guidance, mainly for training the pelvic floor muscles.

**Conclusion:**

Even though they have legal support and access to demand, nurses do not have the knowledge to offer conservative treatment for Lower Urinary Tract Dysfunction. Despite this, they were motivated to do so as long as they received specific training.

## RESUMO

**Objetivo:**

Compreender a atuação dos enfermeiros da Atenção Primaria à Saúde no tratamento da Disfunção do Trato Urinário Inferior.

**Método:**

Pesquisa transversal multi-metodológica, composta por etapas quantitativa e qualitativa, de forma independente e sequencial. Dados coletados de forma remota, por meio de questionário e grupo focal, analisados por estatística descritiva e análise temática de Braun e Clarke, respectivamente. O projeto foi aprovado sob Parecer no. 22691119.0.0000.0030.

**Resultados:**

Participaram do estudo 145 enfermeiros na etapa quantitativa e 20 na qualitativa, atuantes na Atenção Primária à Saúde do Brasil. Dos 93,1% enfermeiros que referiram já terem atendido pessoas com Disfunção do Trato Urinário, apenas 54,4% prestaram orientações, sendo principalmente para treinamento da musculatura do assoalho pélvico.

**Conclusão:**

Mesmo possuindo respaldo legal e acesso à demanda, os enfermeiros não têm conhecimento para oferecer tratamento conservador para Disfunção do Trato Urinário Inferior. Apesar disso, mostraram-se motivados para tal atuação desde que recebam capacitação específica.

## RESUMEN

**Objetivo:**

Comprender el papel del enfermero de Atención Primaria de Salud en el tratamiento de la Disfunción del Tracto Urinario Inferior.

**Método:**

Investigación transversal multimetodológica, compuesta por etapas cuantitativas y cualitativas, de forma independiente y secuencial. Datos recopilados de forma remota, a través de un cuestionario y un grupo focal, analizados mediante estadística descriptiva y análisis temático de Braun y Clarke, respectivamente. El proyecto fue aprobado bajo Opinión 22691119.0.0000.0030.

**Resultados:**

Participaron del estudio 145 enfermeros en la etapa cuantitativa y 20 en la cualitativa, actuando en la Atención Primaria de Salud en Brasil. Del 93,1% de los enfermeros que afirmaron haber atendido ya a personas con Disfunción del Tracto Urinario, sólo el 54,4% brindó orientación, principalmente para el entrenamiento de los músculos del suelo pélvico.

**Conclusión:**

Incluso con apoyo legal y acceso a la demanda, los enfermeros no tienen el conocimiento para ofrecer tratamiento conservador para la Disfunción del Tracto Urinario Inferior. Pese a ello, estaban motivados para hacerlo siempre que recibieran una formación específica.

## INTRODUCTION

Urinary incontinence (UI) is defined as involuntary loss of urine at any frequency or volume, affecting men and women of all ages and with a prevalence that exceeds 35% in the general population. It can occur due to loss of function of the pelvic floor muscles (PFM) or bladder overactivity, more specifically the detrusor muscle^(1)^. In addition to UI, which is classified as a dysfunction in itself or as a symptom that represents storage failure, other symptoms can affect the lower urinary tract, such as increased urinary frequency, urinary urgency, or symptoms that represent bladder emptying failure, such as weak jet or urination effort. When a group of lower urinary tract symptoms has clinical relevance or impacts quality of life (QoL), it is called Lower Urinary Tract Dysfunction (LUTD)^(2)^.

QoL is substantially affected by LUTD and has a strong association with mental health disorders, especially depression. In older adults, it leads to an increased risk of falling and, consequently, increased mortality^(3, 4, 5)^. When related to incomplete emptying of the bladder, LUTD can result in recurrent urinary tract infections or even impaired kidney function^(6)^.

Although highly prevalent and with disastrous impacts, LUTD is neglected by the health system in Brazil and around the world. Firstly, because it is a stigmatizing condition, people who experience it go to great lengths to hide it, especially in the presence of urinary leakage^(7)^. The population does not have access to information, nor the number of people experiencing this situation, nor prevention measures nor first-line treatment. Typically, people seek help when symptoms have become unbearable and the desire for resolution outweighs the shame of exposing the issue. At this time of initial consultation, in the Brazilian Health System (SUS – *Sistema Único de Saúde*), nothing is systematically offered as treatment. The common approach is referral to the secondary and even tertiary level, with long waiting lists and surgical or drug approaches, which are often unnecessary^(8,9)^.

Conservative treatment is defined as a first-line approach for LUTD, consisting of behavioral modifications and PFM training^(1)^. It should be offered to everyone who shows symptoms, even if it requires more invasive measures. These are low-cost and highly effective measures^(10,11)^. The first line of LUTD treatment can be applied in Primary Health Care (PHC), and this practice has proven to be cost-effective in countries that have adopted it^(12,13)^.

In the SUS, PHC is the primary level of health, being considered users’ preferred gateway to care, and nurses can be professionals responsible for early identification of cases and initial treatment, being considered professionals with better technical and human skills for such an approach^(7–9)^.

In Brazil, nurses have legal support to act in the conservative treatment of UI and anal incontinence^(14)^. But contradictorily, the topic is not part of the curriculum of undergraduate courses on a mandatory basis. Thus, understanding the scenario of interest serves as a basis for the subsequent development of an educational program to train PHC nurses to treat LUTD. To this end, the present study aimed to understand PHC nurses’ role in treating patients with LUTD.

## METHOD

### Study Design

This is a multi-methodological study with a cross-sectional design, consisting of a quantitative and a qualitative step, independently and sequentially, to provide an expanded understanding of the phenomenon analyzed and support future solutions in the face of complexity in the health care scenario^(15)^.

### Site

The study was carried out online and, therefore, is not linked to a physical location, but is associated with the place where participants work, comprising PHC in the five regions of Brazil.

### Sample and Selection Criteria

The sample was made up of nurses working in PHC, which was the inclusion criterion. To bring these people together and explain the context of the research, a synchronous online workshop was operationalized and disseminated through *Instituto Fluir*, Civil Society Organization founded by the researchers to disseminate prevention and treatment measures for LUTD to the population and train nurses to work in the area. For wide dissemination, the snowball technique was used, starting with the contacts of PHC nurses who were already in contact with *Instituto Fluir*.

### Data Collection

Data collection took place in June and July 2020 so that the nurses who agreed to participate in the study, by reading and digitally signing the Informed Consent Form, were organized into five WhatsApp^®^ groups of according to the region of the country in which they worked so that they could participate in a workshop and receive an access link to the quantitative step data collection instrument. The workshop took place synchronously online, with the purpose of clarifying the context of the research, its objectives and methodology.

The quantitative step instrument was applied through Google Forms^®^, made available to participants at the end of the workshop, taking an average of 20 minutes to complete it so that, at the end of sending responses, the questionnaire was closed for new shipments. This instrument consisted of 29 questions referring to: sociodemographic characterization; time since training and working in PHC; professional training and performance; extra-professional activities; performance in LUTD; perception regarding potential and barriers to action; and suggestions for means of training in the area.

At the end of the first step, there were five groups of nurses registered for the workshops; these groups referred to the regions of the country where these nurses worked. For the qualitative step, consisting of synchronous online Focus Groups (FG), four nurses were randomly selected from each group (region), using Randomize^®^. In this regard, a group of 20 nurses was formed. Following the recommendations for better conduct of FG^(16)^, the group was divided into two, resulting in two FG with 10 nurses (two per region).

FG sessions were conducted by the main researcher in the role of moderator, and the other four nurses from the research team participated in the role of observers, being previously trained to take notes regarding non-verbal issues, such as periods of silence or facial expressions. The guiding questions of the discussion were: given what you had access to in the workshop, regarding nurses’ role in LUTD, do you consider this role in PHC in the current model possible? What would be the factors that would limit such action? The first FG lasted one hour and twenty-six minutes and the second was one hour and fifty-six minutes. The two meetings held, one for each group, with the purpose of deepening the discussion, were recorded by platform resources and archived in the cloud with restricted access for later transcription and analysis.

### Data Analysis and Treatment

the quantitative data collected by Google Forms^®^ were transferred to a Microsoft Excel^®^ spreadsheet and analyzed using descriptive statistics using Stata/SE v.14.1. StataCorp LP, USA. Quantitative variables were described by mean, standard deviation, medians, minimum and maximum. For categorical variables, frequencies and percentages were presented.

The qualitative data obtained in FG were transcribed, transferred to a Microsoft Excel^®^ spreadsheet, and analyzed using the Braun and Clarke^(17)^ thematic analysis method, following the steps: familiarizing oneself with the topic; generating initial codes; searching for topics; reviewing topics; defining and naming topics; producing the report^(17)^. All steps were conducted by the lead researcher and another nurse from the group.

In step 1 (familiarizing yourself with the topic), FG recordings were fully transcribed into a Microsoft Excel^®^ spreadsheet so that each group composed a tab of the spreadsheet and each speech was transcribed in a cell. In step 2 (generating initial codes), each participant’s speech cell gained a parallel cell with all the codes referring to that speech. In step 3 (searching for topics), in an additional tab, similar codes were grouped by columns named with a potential topic.

In step 4 (revisiting the topics), the codes in each column were subdivided into smaller groups, with the creation of possible subtopics. This action was adopted to facilitate the choice of meaningful speeches that brought together all the group’s views on the same topic. All the statements that generated codes were re-read, and those that best represented the group’s idea were chosen. In step 5 (defining and naming topics), from the subdivision with the excerpt of speeches, topics and subtopics were regrouped into major topics.

In step 6 (producing the report), a table was created with two columns: one for the presentation of the major topics and the other for representative statements that supported them. Therefore, the rigor of selecting speeches that made up all the subtopics of the previous step was followed as well as participants from all regions, preserving the representativeness of speeches.

### Ethical Aspects

The project was approved under Opinion 22691119.0.0000.0030 of 06/10/2020, in line with Resolution 466/12 of the Brazilian National Health Council, as it involves human beings. To ensure anonymity, in the quantitative step, no name was linked, and in the qualitative step, participants were coded by their region of activity in the national territory and their order of participation in FG (e.g., Nurse South 01).

## RESULTS

A total of 145 nurses working at PHC in Brazil participated in the quantitative step. The mean age was 37.5 years (SD 7.4). Regarding gender, 130 (89.7%) were women, with the majority of the sample (62.5%) in a stable union.

As shown in Table 1, nurses from 20 Brazilian states participated. The state with the highest number of participants was Rio de Janeiro, with 28 nurses. The states of Rio Grande do Norte, Sergipe and Pernambuco had the participation of a nurse.

**Table 1 –:** Distribution of Primary Health Care nurses participating in the research by region and state – Brasília, Federal District, Brazil, 2021.

Region and states	N	%
**North**	**10**	**6.9**
Acre	2	1.4
Amazonas	4	2.8
Rondônia	4	2.8
**Northeast**	**33**	**22.8**
Bahia	5	3.4
Ceará	7	4.8
Maranhão	3	2.1
Pernambuco	1	0.7
Piauí	15	10.3
Rio Grande do Norte	1	0.7
Sergipe	1	0.7
**Center-West**	**31**	**21.4**
Distrito Federal	17	11.7
Goiás	2	1.4
Mato Grosso do Sul	12	8.3
**Southeast**	**44**	**30.3**
Espírito Santo	2	1.4
Minas Gerais	4	2.8
Rio de Janeiro	28	19.3
São Paulo	10	6.9
**Sul**	**27**	**18.6**
Paraná	10	6.9
Rio Grande do Sul	9	6.2
Santa Catarina	8	5.5
**Total**	**145**	**100**

Nurses’ mean training time was 11.8 years (SD 7.1) with a variation between 0.5 and 34 years. Ninety-three participants (64.1%) had more than 10 years of training and only 11 (7.6%) had less than two years. Nineteen (13%) had other higher education, and 129 (89%) had a *lato sensu* graduate degree. The predominant area was collective health (n. 80 *–* 55.8%), followed by obstetrics (n. 25 *–* 17.1%) and stoma therapy (n. 25 *–* 17.1%). Twenty-five participants (17%) had a master’s or doctorate and 76 (51.9%) completed two or more *lato sensu* graduate courses. Thirty-six (25%) had other employment relationships besides PHC.

The majority of the sample (n. 135 *–* 93.1%) had already treated people with LUTD, and, of these, 66 (48.8%) said they had already offered some guidance, although without adequate knowledge. It can be seen in Table 2 that the predominant guidance was for pelvic floor muscle training, followed by guidance for clean intermittent catheterization.

**Table 2 –:** Activities carried out in the care of people with Lower Urinary Tract Dysfunction by Primary Health Care nurses participating in the research – Brasilia, Federal District, Brazil, 2021.

Activity or guidance	N	%^*^
Pelvic floor strengthening exercises	50	75.8
Clean intermittent catheterization	13	19.7
Guidance for water intake	8	12.1
Care with indwelling bladder catheterization	5	7.6
Guidance on reducing potential bladder irritants	4	6.1
Urinary frequency control	3	4.5
Vaginal pessary	1	1.5

^*^Percentages calculated on the total number of participants who stated that they had carried out an activity or guidance and answered what they were (n = 66).

When asked what would prevent them from working to care for people with LUTD if they had the knowledge to do so, 52 (36.1%) responded that nothing would stop them and 29 (20.2%) still felt that they might find their knowledge insufficient or feel insecurity in care, and the same percentage indicated that the issue of time, demand and schedule could be an impediment. The complete data is presented in Table 3. These questions were deepened in FG, described in the qualitative step.

**Table 3 –:** Primary Health Care nurses’ perception regarding what would prevent them from working on Lower Urinary Tract Dysfunction if they had the knowledge – Brasília, Federal District, Brazil, 2021.

Impediment	n	%^*^
Nothing	43	36.1
Time/demand/schedule	24	20.2
Insecurity/insufficient knowledge	24	20.2
Lack of physical or material resources	16	13.4
Patient non-acceptance	9	7.6
Lack of management support	7	5.9
Lack of support from the class council	3	2.5
Lack of protocol	3	2.5

^*^Percentages calculated from the total number of participants who answered the question about what would prevent them from applying the knowledge acquired (n = 119).

There was no statistically significant difference in the association of variables related to impediment factors for working in LUTD, when analyzed, according to the region of the country where nurses worked.

A total of 20 nurses participated in the qualitative step, divided into two groups of 10, with two representatives from each region of the country in each group. As a result of thematic analysis, the initial code grouping resulted in 17 topics. The topic with the lowest number of codes was “approaching the LUTD topic in PHC”, with six codes, and the largest number of codes was for the “service perspectives” topic, with 68 codes, as shown in Table 4.

**Table 4 –:** Initial topics and frequency of codes according to thematic analysis of Focus Group “Action of Primary Health Care Nurses in Lower Urinary Tract Dysfunction” – Brasília, Federal District, Brazil, 2021.

Initial topics	Number of codes
Previous knowledge on the topic	14
Previous workshop experience	7
Demand/possibilities of action	35
Management of LUTD cases at PHC	17
Perception regarding nurses’ role today	7
Approach to the topic in graduation	8
Approach to the topic in graduate studies	8
Approach to the topic in PHC practice	
Perception regarding the possibilities for nurses	28
Emotions and feelings regarding the topic	26
Workshop assessment	32
Service perspectives	68
Relationship with the team	14
Relationship with the manager	9
Relationship with the population	8
Fears/uncertainties/challenges/limitations	34
Schedule/demand/overload	13

LUTD - Lower Urinary Tract Dysfunction; PHC - Primary Health Care.

After dividing the topics into subtopics, choosing representative statements for each subtopic and completely rereading the material, the final grouping was carried out into three major topics: Lack of knowledge regarding nurses’ role in LUTD; Previous experience in caring for people with LUTD; and Perceptions based on nurses’ proposed role in PHC.

Some statements were noted in the narratives that represent the distribution of participants between regions and training time, in the same way that the quantitative step demonstrated:

*I’m here from Porto Velho, Rondônia. I’ve been graduating for a while now, and I’ve been working in Primary Care all this time, about 22 years. (Nur North 02)*

*I work in a Family Clinic in the city of Rio. I have worked in primary care since 2011, in the same unit, on the same team. (Nur Southeast 01)*

*I’m from Minas Gerais, but I live here in Brasília, FD. I am a nursing resident through Fiocruz Brasília, I graduated in December 2019. I am still in care. My first experience. (Nur Center-West 02)*

In the “Lack of knowledge regarding nurses’ role in LUTD” topic, the narratives grouped under this topic reveal a gap in knowledge that was not offered to them during their undergraduate course, in *lato sensu* graduate courses or in care practice in PHC:

*I don’t remember when I graduated there was any activity scheduled, a nursing consultation focused on incontinence, right? (Nur South 02).*

*I’ve been in this service for a few years, which is a reference place for various Primary Care issues in Brazil, and this topic has never been addressed (Nur South 02).*

*There are a number of other nurses who have no idea how nurses can contribute. How could I imagine that nurses can contribute to urinary incontinence treatment? (Nur Northeast 04).*

In the “Previous experiences in caring for people with LUTD” topic, it is observed that nurses notice complaints of symptoms in care practice, but the frequent action was to refer them to urology or gynecology or physiotherapy specialties. UI treatment does not even make up the classification of nursing practices in PHC:

*I was actually taking a look at nursing classification at PHC. We even have urinary incontinence, but I don’t see the treatment part, it’s not incorporated (Nur Northeast 01).*

*That’s where we refer, either physiotherapy or urologist, gynecologist (Nur South 03).*

*And it was always like this, referrals to the gynecologist, a lot of surgical intervention, things that we know could happen. Wow, with this course, I’m sure it will change a lot (Nur North 02).*

In the “Perceptions based on nurses’ proposed role in PHC” topic, it is echoed that, after having had contact with the topic and understood this possibility of action, participants expressed a perception that it was possible for nurses to treat cases of LUTD in PHC, demonstrating surprise and happiness, citing the empowerment that the category can achieve with this practice and highlighting the simplicity of actions that involve more knowledge than resources.

*It is a very vast field, yes, for nurses. I think we can work very well on this in Primary Care (Nur Center-West 04)*

*We see that these are simple actions. We can contribute to people’s lives, to their quality of life (Nur Northeast 01).*

*Jesus, I keep thinking. When I saw the amount of field of action, of how we can empower ourselves as a class, I was super happy, you know? (Nur North 02).*

*So, I believe it was a, I think it was like an awakening, like, right, I’m going to read more, I’m going to try to multiply it with my co-workers, right? (Nur South 02).*

*We can become a multiplier of this knowledge, passing it on and further expanding the engagement of other professionals (Nur Northeast 02).*

*I think everyone could try to fit this into the life of a PHC nurse. Like the manager’s approval, of course! That you can see, that your eyes shine, seeing this as important too (Nur Southeast 02).*

## DISCUSSION

This study aimed to understand PHC nurses’ role in treating patients with LUTD. It is noteworthy that, although most of the sample worked at PHC for more than 10 years and had more than one *lato sensu* graduate course, they did not have access to LUTD content throughout their training or professional life. Almost the entire sample had treated patients with LUTD, whether with manifestations of UI, urinary urgency or symptoms of incomplete bladder emptying. Even without having received any targeted training, more than half of these nurses provided some guidance to patients with LUTD, with the predominant guidance being for PFM training. There were very few cases of guidance for behavioral changes, such as improving water intake, positioning for elimination or maneuvers to inhibit urgency. It is worth mentioning that, when mentioning guidance for PFM, nurses referred to telling patients that pelvic contraction and relaxation movements could be performed a few times a day. No nurses performed functional assessment or included patients in progressive training programs.

Similar results were observed in a cross-sectional study carried out in China regarding a tendency towards positive attitude and practice as opposed to an important gap in knowledge. 1,313 students from six nursing faculties participated. It was observed that general knowledge about UI ranged from 15 to 30%, while positive attitude (what they believe about the topic) was from 43 to 60%, and a high level of interest in learning more about the topic was observed^(18)^.

The same setting seems to be repeated too, but among professionals. A cross-sectional study conducted in two public hospitals and four private hospitals in Turkey assessed the knowledge and attitude of 254 nurses regarding UI. Similarly, the score for attitude was higher in relation to knowledge, reaching an average of 46 (15*–*60) and 15 (0*–*24), respectively^(19)^.

The discussion in the FG reinforced the evidence that nurses at Brazilian PHC do not have training to care for people with LUTD, and participants mention not having had contact with the topic during their graduation. In this context, research published in 2020 assessed knowledge regarding UI of 1,581 final-year students from the schools of nursing, medicine and physiotherapy. Nursing students scored 88.8% correctly, medicine students scored 81.7%, and physiotherapy students scored 74.4% correctly^(20)^. Even though the aforementioned study was carried out in Poland, a country with a different reality from Brazil, knowledge of future professionals regarding the topic may sound like an evolution, since the nurses participating in this study had no contact with the topic during their undergraduate studies. On the other hand, it is worth highlighting that adequate knowledge will not necessarily result in adequate practice. For knowledge to be transformed into practice, organized actions are necessary that involve analysis of available evidence, the context in which it will be applied and facilitation strategies for implementation^(21)^. Elucidating this statement, a study carried out in China observed that 80% of obstetric nurses knew and believed that PFM training was effective in perinatal UI prevention and treatment, but less than 30% advised patients to perform it^(22)^.

It is worth noting that nurses’ role is presented as one of the promising actions to reduce LUTD rates, especially in PHC^(23, 24, 25)^. In this context, a study conducted in China assessed the effectiveness of a treatment program for adults with LUTD through the intervention of PHC nurses from PFM training, bladder training, water control, reducing the consumption of potential bladder irritants and urethral massage to control post-void dripping. Significant improvements were noted in symptom severity, overall health, reduction in medical appointments and medication use^(13)^.

The motivation that the topic is capable of generating in nurses was observed as relevant data. The highest percentage of participants said that nothing would stop them from working in LUTD if they had knowledge. In FG, the participating nurses expressed surprise and positive expectations when they learned about the legal support that nurses have to work in the area, in addition to the autonomy and resoluteness of their actions, which are simple and low cost.

International authors argue that nurses are in a leading position to help people affected by UI, with potential for identification, health education and implementation of appropriate interventions. They mention that this professional’s position is ideal for carrying out initial assessment and treatment, important steps of care, but which are generally poorly executed^(7,26)^.

Given the participants’ expression of surprise at actions’ simplicity and low cost, it is necessary to reflect on economic aspects related to PHC nurses’ role in LUTD. In addition to the personal impact that LUTD has on the individuals who experience it, the economic impact is also relevant in several aspects: waiting lines for specialized services in secondary and tertiary care centers are occupied by people who could have been treated in PHC; thus, access for people who are in real need of these levels of care is hampered. Delays in identifying complicated or recurrent cases can lead to their worsening, which can lead to loss of kidney function, burdening the system with high-cost diagnostic tests and interventions. The lack of supply for conservative treatment of LUTD in PHC results in excessive and unnecessary indications for surgical procedures, consuming resources not only for the procedure itself, but also for the tests involved in the process, as is the case with urodynamic studies.

In this economic context, a study carried out in the Netherlands analyzed the cost-effectiveness of including a nurse specializing in incontinence in PHC, with a view to increasing the detection and early intervention of UI cases. Through a decision analytical model, which compared the results of the previous structure with the new proposal, savings of 402 euros per patient were observed over a period of three months^(27)^.

As limitations of this study, it is noteworthy that, although the invitation to participate in the study was open to any nurses working in PHC in Brazil, those who participated in the quantitative phase of the study were not equally representative of the five regions. On the other hand, the importance of this study is echoed, which elucidated nurses’ role in treating LUTD in PHC, and could be a precursor for future research with larger samples, but above all to support actions aimed at strengthening nursing practice in this context.

## CONCLUSION

The study results elucidate that, despite legal support and being a significant demand in PHC, nurses were not aware of their possibilities of action in LUTD prevention and treatment, and had not had contact with the topic in their training. Despite this, the majority indicated that, if they had knowledge in the area, nothing would stop them from acting. Of those who mentioned an impediment, they mentioned insufficient knowledge, which could be overcome by training programs, or a question of time, demand and agenda, which would be resolved if care for people with LUTD was prioritized as a PHC action and became part of service goals.

It is expected, through this understanding of nurses’ role in LUTD prevention and treatment in PHC, to subsidize educational programs that equip them to offer conservative treatment to the population with LUTD, in order to reduce injuries, time of exposure to symptoms, waiting lists and unnecessary surgical procedures.
